# Treatment of COVID-19 anxiety by auricular points: A protocol for systematic review and meta-analysis

**DOI:** 10.1097/MD.0000000000028984

**Published:** 2022-03-11

**Authors:** Xingxin Wang, Yulei Zhao, Xinyu Yang, Zhongqi Fan, Ziyue Wang, Ping Zhang, Jun Chen

**Affiliations:** a Shandong University of Traditional Chinese Medicine, Jinan, Shandong, China; b Taishan District People's Hospital of Tai’an City, Tai’an, Shandong, China.

**Keywords:** auricular points, Corona Virus Disease 2019, network meta-analysis, protocol, systematic review, traditional Chinese medicine

## Abstract

**Background:**

In December 2019, a series of acute, atypical respiratory diseases was identified in Wuhan, China. The source of the illnesses was attributed to a novel coronavirus, named the severe acute respiratory syndrome coronavirus-2 (SARS-CoV-2), and the subsequent disease it causes was named the coronavirus disease 2019 (COVID-19). Evidence from previous coronavirus outbreaks has shown that infected patients are at risk for developing psychiatric and mental health disorders, such as depression, anxiety, and sleep disturbances.

**Methods:**

According to the retrieval strategies, randomized controlled trials on auricular points for anxiety in patients with coronavirus 2019 will be obtained from the China National Knowledge Infrastructure, WanFang Data, Chinese Scientific Journals Database, PubMed, Embase, and Cochrane Library, regardless of publication date or language. Studies will be screened based on inclusion and exclusion criteria, and the Cochrane risk bias assessment tool will be used to evaluate the quality of the literature. The network meta-analysis will be performed with the Markov chain Monte Carlo method and carried out with Stata 14.2 and WinBUGS 1.4.3 software. Ultimately, the quality of the evidence obtained from the results will be evaluated.

**Results:**

This study will evaluate whether auricular points can effectively treat anxiety in patients with COVID-19.

**Conclusion:**

This study will provide evidence for whether auricular points is beneficial to the treatment of anxiety in patients with COVID-19.

**INPLASY registration number:**

CRD42022302649.

## 1 Introduction

The medical havoc wreaked by the novel severe acute respiratory syndrome coronavirus (SARS-CoV-2) has grown exponentially to pandemic proportions within a short span of time, starting in December 2019. Researchers in China examined psychological responses during the initial stage of the Corona Virus Disease 2019 (COVID-19) epidemic in the general population.^[1]^ They found that 53.8% of respondents rated the psychological impact of the outbreak as moderate or severe, 16.5% reported moderate to severe depressive symptoms, and 28.8% reported moderate to severe anxiety symptoms. Another nationwide survey of more than 50,000 people in China during the COVID-19 epidemic showed that about 35% of the respondents experienced psychological distress.^[2]^ Another research group in China sampled and analyzed the online posts from about 18,000 Chinese social media users before and after the declaration of COVID-19 in China on January 20, 2020.^[3]^ Researchers found that negative emotions such as anxiety, depression, and anger increased, whereas positive emotions and life satisfaction decreased.

Previous studies suggest that depression, anxiety disorders, substance abuse, increased suicidal tendencies, and PTSD commonly follow major economic crises or natural disasters.^[4–6]^ If similar patterns hold for the COVID-19 pandemic, the psychological effects of persistence stress among the general population and exacerbation of several mental health disorders among the vulnerable individuals will further strain the current health care system. It may also prevent resumption to normal life for many people when the physical threat to viral infection eventually subsides. The disruption of a normal life as a result of a government-imposed lockdown or stay home orders has significantly impacted the mental health of the affected individuals.^[7,8]^ A recent umbrella review of mental health outcomes of quarantine and similar prevention strategies has found that depression, anxiety disorders, mood disorders, posttraumatic stress symptoms, sleep disorders, panic, stigmatization, low self-esteem, lack of self-control are highly prevalent among individuals impacted with physical isolation.^[9]^ Another rapid review suggested that stressors like prolonged quarantine, fear of infection, frustration, boredom, inadequate supplies, inadequate information, financial loss, and stigma have resulted in long-lasting posttraumatic stress symptoms, confusion, and anger in the mass population.^[10]^

Auricular points includes acupuncture, electroacupuncture, acupressure, lasering, cauterization, moxibustion, and bloodletting in the auricle. For 2500 years, people have employed auricular points for treating diseases, but the methods have been limited to bloodletting and cauterization. Only after 1957, the international scientific community became aware that the map of the ear resembles an inverted fetus, its introduction has led to auricular acupuncture (AA) becoming a more systemic approach, and, following the identification and standardization of more precise points, AA has been employed in clinical applications. The mechanisms of AA are considered to have a close relationship with the autonomic nervous system, the neuroendocrine system, neuroimmunological factors, neuroinflammation, and neural reflex, as well as antioxidation. auricular points has been applied, for example, for pain relief, for the treatment of epilepsy, anxiety, and obesity, and for improving sleep quality.^[11]^

In recent years, there are more and more reports on auricular point therapy for COVID-19, but the research on auricular point therapy for anxiety in patients with coronavirus 2019 (C19-A) has not been systematically reviewed. Therefore, we decided to fill the gap in the literature to provide experts and patients with up-to-date evidence that can be used to rigorously evaluate the effectiveness of this therapy and to guide clinical practice. Efficacy and safety of auricular point therapy for C19-A were summarized.

## 2 Methods

### 2.1 Objectives and registration

This systematic review will aim to evaluate the effect and safety of auricular points for C19-A. Our protocol has been registered on the International Platform of Registered Systematic Review and Meta-Analysis Protocols (PROSPERO). The registration number was CRD42022302649. All steps of this systematic review will be performed according to the Cochrane Handbook (5.2.0).

### 2.2 Ethics and dissemination plans

Given that there will be no patients recruited and no data gathered from patients, ethical approval is not necessary for our research. We will publish the results of this network meta-analysis in the form of journal papers or conference papers.

### 2.3 Eligibility criteria

PICOS principles will be consulted to establish the inclusion and exclusion criteria of this systematic review.

#### 2.3.1 Types of participants

The eligible studies will be those who have been assessed as covid-19 patients with anxiety, without any age, gender or racial restrictions.

#### 2.3.2 Types of interventions and comparators

Auricular points includes acupuncture, electroacupuncture, acupressure, lasering, cauterization, moxibustion, and bloodletting in the auricle. Studies that combine auricular points with other therapies, such as acupuncture, massage, drugs, and physical interventions, will be included if they can prove that auricular points is effective.

#### 2.3.3 Types of outcomes

The primary outcomes included the effective rate of clinical symptoms, Curative effect and length of hospital stay of pneumonia, and self-rating anxiety scale. The secondary outcomes will assess health evaluation and the incidence of adverse events.

#### 2.3.4 Types of studies

The selected articles should be randomized controlled trials comparing auricular therapy and control groups to evaluate the efficacy of auricular points on C19-A. We will include an assessment of auricular points compared with control interventions, including inactive controls (such as placebo, no treatment) and active controls (such as drugs and acupuncture). Conference literature and papers, reviews, case series, case reports, experience summaries, and animal research will be excluded.

### 2.4 Data sources and retrieval strategy

We will search foreign and Chinese databases, including PubMed, EMBASE, MEDLINE, CENTRAL, CNKI, WanFang Data, CBM, and VIP from the inception of the coverage of these databases to July 2021.

Data, CBM, and VIP from the inception of the coverage of these databases to July 2020. The databases will be retrieved by combining the subject words with random words. Taking PubMed as an example, the retrieval strategy is shown in Table 1.

**Table 1:** PubMed search strategy.

Number	Search items
#1	“anxiety”[MeSH]
#2	“anxiety”[Title/Abstract] OR “Angst”[Title/Abstract] OR “Social Anxiety”[Title/Abstract] OR “Anxieties, Social”[Title/Abstract] OR “Anxiety,Social”[Title/Abstract] OR “Social Anxieties”[Title/Abstract] OR “Hypervigilance”[Title/Abstract]OR “Nervousness”[Title/Abstract] OR “Anxiousness”[Title/Abstract].
#3	“COVID-19”[Title/Abstract].
#4	“auricular points”[title/abstract] or “auricular therapy”[title/abstract] or “acupuncture”[title/abstract] or “electroacupuncture”[title/abstract] or “acupressure”[title/abstract] or “lasering”[title/abstract]. or “cauterization”[title/abstract] or “moxibustion”[title/abstract] or “bloodletting”[title/abstract].
#5	“randomized controlled trial”[Title/Abstract] OR “randomized”[Title/Abstract] OR “placebo”[Title/Abstract].
#6	#2 and #3 and #4 and #5

The search terms will be adapted appropriately to conform to the different syntax rules of the different databases.

### 2.5 Study selection and data extraction

**Figure 1:**
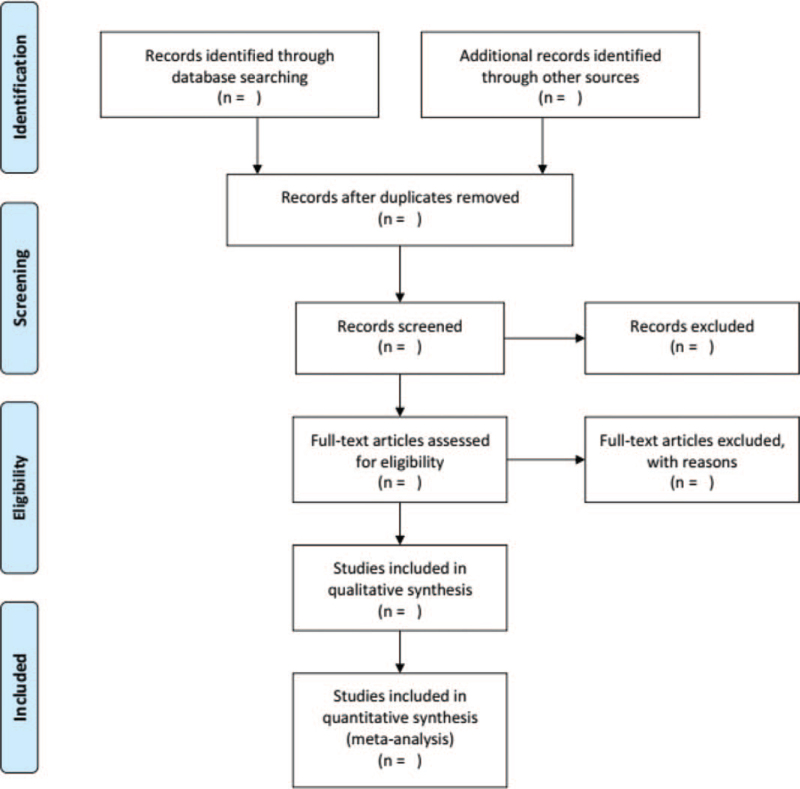
PRISMA flow chart.

EndNote X9 will be used to manage the retrieved studies. As shown in Figure 1, the study selection will be divided into 2 steps and completed by 2 researchers (XXW and LD). Preliminary screening: Duplicate and irrelevant studies will be deleted while screening the titles and abstracts. Rescreening: We read through the full texts and select studies according to the inclusion and exclusion criteria.

According to the Cochrane Handbook for Systematic Reviews of Interventions, the 2 researchers (XXW and LD) will extract data, including the author, publication time, participant number, age, race, lesion location, intervention measures, course of treatment and outcome indicators, and they will enter these data in the data extraction table to compare results.

### 2.6 Risk of bias assessment

Two researchers (XXW and YH) will assess the quality of the included clinical randomized controlled trials independently by utilizing the Cochrane Risk of Bias assessment tool. As specified by the Cochrane Handbook (5.2.0), the following sources of bias will be considered: random sequence generation, allocation concealment, participant blinding, outcome assessor blinding, incomplete outcome data, selective reporting, and other sources of bias. Each domain will be rated as having a high, low, or unclear risk of bias as appropriate.^[12]^ The 2 reviewers will resolve any disagreements through discussion, and a third reviewer (LD) will be consulted if no consensus is reached.

### 2.7 Statistical analysis

#### 2.7.1 Traditional meta-analysis

Direct comparisons of auricular points efficacy will be performed using Review Manager 5.3. The outcomes will be mainly represented by the mean difference or odds ratio with 95% confidence intervals, and a *P* value < .05 will be considered significant. The Cochrane Q test and *I*^2^ statistics will be used to assess heterogeneity. When *P* < .1 or *I*^2^ > 50%, which indicates statistical heterogeneity, a random effects model will be used to calculate the outcomes; otherwise, a fixed effects model will be considered.

#### 2.7.2 Network meta-analysis

A network evidence diagram will be drawn to visually represent the comparisons between the studies. The size of the nodes represents the number of participants, and the thickness of the edges represents the number of comparisons. Stata 14.2 and WinBUGS 1.4.3 Software will be used to carry out Bayesian network meta-analysis. Bayesian inference will be carried out using the Markov chain Monte Carlo method, the posterior probability will be inferred from the prior probability, and estimation and inference will be assumed when Markov chain Monte Carlo reaches a stable convergence state. As a result, the rank of the auricular points effect will be presented by the surface under the cumulative ranking curve.

Inconsistencies between direct and indirect comparisons will be evaluated using the node splitting method.^[13]^ The choices between fixed effects and random effect models and between consistent and inconsistent models will be made by comparing the deviance information criteria for each model.^[14,15]^

#### 2.7.3 Subgroup and sensitivity analysis

If the heterogeneity is high, we will also perform subgroup analysis to calculate the combined statistics.^[16]^ The following subgroup analyses will be considered: gender, age, intervention time, intervention cycle, and course of the disease.

When sufficient data are available, sensitivity analysis will be performed to test the robustness of the primary outcomes, which includes assessing the quality of the methods, the quality of the studies, and the impact of sample size and missing data.

#### 2.7.4 Publication biases

If 10 or more studies are included, we will use funnel plots to assess the level of publication bias. Asymmetry in the funnel plot will suggest the possibility of small study effects, and the results of the analysis will be interpreted cautiously.

### 2.8 Quality of evidence

The Grading of Recommendations Assessment, Development and Evaluation (GRADE) system will be used to assess the overall quality of the evidence derived from the included studies.^[16]^ In addition, the results will be divided into high, moderate, low, and very low quality.

## 3 Discussion

Auricular points, a very ancient modality of treating diseases, has been used throughout the history of human civilization and plays an important role in disease resistance. Auricular points has been widely used for various conditions, including cancer, ulcerative colitis, stroke rehabilitation, constipation, hypertension, pain conditions and breech presentation. At present, auricular points are used to treat anxiety in patients with COVID-19, but there is no systematic study to provide evidence that auricular points are effective in the treatment of C19-A. We hope that the results of this study can provide a basis for auricular points treatment of C19-A.

## Author contributions

**Conceptualization:** Xingxin Wang, Zhongqi Fan, Jun Chen.

**Data curation:** Xingxin Wang, Yulei Zhao, Ziyue Wang, Ping Zhang, Xinyu Yang, Jun Chen.

**Formal analysis:** Xingxin Wang and Zhongqi Fan.

**Methodology**: Jun Chen.

**Software:** Zhongqi Fan and Yulei Zhao

**Supervision:** Jun Chen.

**Writing – original draft:** Xingxin Wang.

**Writing – review & editing:** Jun Chen.
